# Using the Life Story Book With Mentally Alert Residents of Nursing Homes

**DOI:** 10.1093/geroni/igaa057.1574

**Published:** 2020-12-16

**Authors:** Theresa Chrisman

**Affiliations:** University of Houston, Houston, Texas, United States

## Abstract

Depression and lack of meaning in life (MIL) are common among residents of nursing homes (NHs) and contribute to a reduction in overall health and well-being. Life Story Book (LSB), a reminiscence intervention, is designed to provide a person with the opportunity to review their past and capture their life stories and photographs into a book. LSB has demonstrated positive outcomes for residents of NHs with dementia, yet little is known for residents without dementia. A switching replication design was used to examine the effects of LSB among 21 mentally alert residents from two NHs (NH-A and NH-B) in Houston, Texas. Participants in NH-A received three weeks of the LSB intervention, while NH-B received three weeks of care-as-usual; the intervention was then switched. The GDS-12R and the MIL questionnaire (MLQ) were used to measure depressive symptoms and MIL respectively. Participants from NH-A (n =11) and NH-B (n = 10) had a mean age of 75 years (SD =11.34); 81% female; 52% non-Hispanic white and 33% African American. Results from a one-way MANCOVA found no statistically significant difference on the GDS-12R and MLQ (F(3, 14) = 2.50, p = .102; Wilks’ Lambda = .652; η2 = .35). Further analyses comparing the pre-intervention and post-intervention scores for the entire sample (N =21) found a significant reduction in depressive symptoms (M = 2.67; SD = 2.52) and (M =1.67, SD = 2.29); (t (20) = 2.21, p = 0.039). The potential benefits of LSB for mentally alert residents of NHs warrants further research.

